# Effectiveness of joint mobilisation after cast immobilisation for ankle fracture: a protocol for a randomised controlled trial [ACTRN012605000143628]

**DOI:** 10.1186/1471-2474-7-46

**Published:** 2006-05-26

**Authors:** C Christine Lin, Anne M Moseley, Kathryn M Refshauge, Marion Haas, Robert D Herbert

**Affiliations:** 1 School of Physiotherapy, the University of Sydney, PO Box 170, Lidcombe, New South Wales 1825, Australia; 2 Centre for Health Economics Research and Evaluation (CHERE), University of Technology, Sydney, PO Box 123, Broadway, New South Wales 2007, Australia

## Abstract

**Background:**

Passive joint mobilisation is a technique frequently used by physiotherapists to reduce pain, improve joint movement and facilitate a return to activities after injury, but its use after ankle fracture is currently based on limited evidence. The primary aim of this trial is to determine if adding joint mobilisation to a standard exercise programme is effective and cost-effective after cast immobilisation for ankle fracture in adults.

**Methods/Design:**

Ninety participants will be recruited from the physiotherapy departments of three teaching hospitals and randomly allocated to treatment or control groups using a concealed procedure. All participants will perform an exercise programme. Participants in the treatment group will also receive joint mobilisation twice a week for four weeks. Blinded follow-up assessments will be conducted four, 12 and 24 weeks after randomisation. The primary outcome measures will be the Lower Extremity Functional Scale and the Assessment of Quality of Life. Secondary outcomes will include measures of impairments, activity limitation and participation. Data on the use of physiotherapy services and participants' out-of-pocket costs will be collected for the cost-effective and cost-utility analyses. To test the effects of treatment, between-group differences will be examined with analysis of covariance using a regression approach. The primary conclusions will be based on the four-week follow-up data.

**Discussion:**

This trial incorporates features known to minimise bias. It uses a pragmatic design to reflect clinical practice and maximise generalisability. Results from this trial will contribute to an evidence-based approach for rehabilitation after ankle fracture.

## Background

Ankle fracture refers to fracture of the medial or lateral malleolus or the distal tibia or fibula [1-4]. The incidence is between 107 and 184 per 100,000 person-years [5-8], making it one of the most common lower limb fractures [9,10]. It is usually caused by low-velocity trauma such as falls, twisting injuries and sports injuries [6-8], and therefore is prevalent in not only the older, but also the young and active population [6,8]. Orthopaedic management in adults with ankle fracture may involve reduction, surgical fixation, and immobilisation for six to 12 weeks depending on the severity of the fracture [3,4,11-13]. Not surprisingly, fracture severity has been reported to influence functional outcomes after ankle fracture [9,14,15].

Physiotherapists often contribute to the rehabilitation of ankle fracture, which typically starts soon after the period of immobilisation. Due to effects of the fracture and the subsequent immobilisation, most people experience pain, swelling, stiffness, muscle atrophy and decreased muscle torque at the ankle following cast removal [16-19]. Consequently, they complain of limitations in activities involving the lower limb, such as stair climbing and walking [18], and reduced participation in work and recreation [20-22].

Passive joint mobilisation is a technique commonly used by physiotherapists to address the problems of pain and joint stiffness, in order to allow an earlier return to activities. It involves the physiotherapist manually gliding the articular surfaces of a joint to produce oscillatory movements [23]. It has been proposed that manual therapy such as joint mobilisation produces analgesic effects and increases elasticity of joint structures through interactions at the local, central nervous system and psychological levels [24]. These hypotheses are yet to be validated [25]. Research to-date indicates that the analgesic effect of peripheral joint mobilisation may be short lasting [26], is not mediated by endorphins [27], and can been seen with concurrent excitation of the sympathetic nervous system [28,29].

The effectiveness of joint mobilisation after post-fracture immobilisation has been investigated in a small number of trials with contrasting results. Randall et al [30] showed that in patients with metacarpophalangeal fracture, mobilisation and exercise led to a greater increase in joint movement than exercise alone. Coyle and Robertson [31] reported that mobilisation of the wrist improved pain and extension movement after Colles' fracture, but the results were not compared to a control group. Two other studies of mobilisation after Colles' fracture found no significant differences between outcomes of treatment and control groups [32,33].

There is positive evidence to support the use of joint mobilisation after acute ankle sprain [34,35]. Joint mobilisation led to increased ankle movement, improved gait patterns, reduced pain and the number of treatment sessions required, and hastened return to activities when compared with the usual rest, ice, compression and elevation protocol [35]. However, the effectiveness of joint mobilisation after ankle fracture is less certain. Wilson [36] conducted a randomised pilot study to investigate if joint mobilisation plus whirlpool therapy and exercise was more effective than whirlpool therapy and exercise alone. Although an improvement in ankle movement and function was reported when the treatment group was compared to the control group, the difference between groups was not statistically significant, potentially because of the small sample size (n = 10).

The treatment cost of ankle fracture has been estimated in studies from Britain and Canada [37,38], but to-date there are no randomised controlled trials. In a retrospective audit, costs of hospitalisation were significantly greater in those who were operated on within 24 hours, compared to those who were operated on more than 24 hours after presentation [37]. The average extra cost per patient was £990 (1998 British pounds). A prospective pilot study reported that the total hospitalisation and outpatient medical costs were $2,134 (United States dollars) per patient over a 12-month period [38]. But this study did not include a control group, and hence no comparisons of costs or quality of life measures. Neither of these studies assessed utilisation of services or included the cost of outpatient physiotherapy. Only one published study has reported an economic analysis of joint mobilisation [39], but the comparisons made were between high-grade and low-grade mobilisation for the shoulder. Therefore, despite the common use of joint mobilisation as a treatment technique, its current use after ankle fracture is based on clinical experience and limited evidence of effectiveness and cost-effectiveness.

The primary aim of this trial is to determine whether adding joint mobilisation to an exercise programme is more effective and cost-effective than exercise alone after cast immobilisation for ankle fracture in adults. A secondary aim is to determine if treatment effects are influenced by fracture severity.

## Methods

### Design

**Figure 1:**
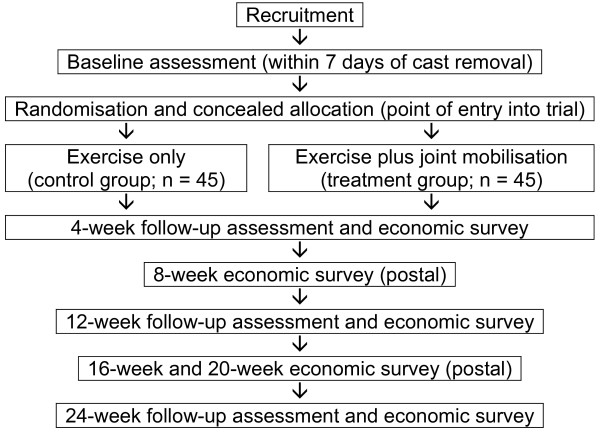
Experimental protocol.

This will be an assessor-blinded, multi-centre, randomised controlled trial (Figure 1). Participants will be stratified by site and randomly allocated in permuted blocks to treatment or control groups. The randomisation sequence will be generated a priori using the random number function in Excel by an independent investigator not directly involved in the assessment and treatment of participants. Allocations will be sealed in opaque and consecutively numbered envelopes, which will be opened in sequence by the treating physiotherapist after the recruitment and baseline assessment of each participant.

### Participants

Participants will be recruited following cast removal from the plaster clinics and physiotherapy departments of three large teaching hospitals in Sydney, Australia (Royal North Shore Hospital, Royal Prince Alfred Hospital, and St Vincent's Public Hospital). The inclusion criteria will be:

• ankle fracture treated with cast immobilisation with or without surgical fixation

• cast removed in the preceding seven days

• approval from the orthopaedic specialist to weight-bear as tolerated or partial weight-bear

• referral to outpatient physiotherapy for treatment

• at least 2 out of 10 pain in the ankle when up to 50% of body weight is borne through the affected leg

• completed skeletal growth assessed by the union of epiphyses on radiographs

• no concurrent and significant injuries or pathologies which may affect the recovery of the participant's lower limb function, and

• available for the six month follow-up period.

In addition to recruiting from multiple sites, other strategies will be used to maximise recruitment rates [40]. A log will be designed to aid the screening of potential participants at each site, and will be monitored by the investigators. Posters about the trial, inservices and regular communication with the participating physiotherapy departments will also assist to facilitate recruitment rates.

Ethical approval will be obtained from the ethics committee of each participating site (i.e., the University of Sydney Human Research Ethics Committee, Northern Sydney Health Human Research Ethics Committee, Sydney South West Area Health Service Ethics Review Committee (RPAH zone), and the St Vincent's Hospital Human Research Ethics Committee).

### Interventions

Interventions will be delivered by registered physiotherapists, who will receive training to provide treatments in accordance with the experimental protocol.

**Figure 2:**
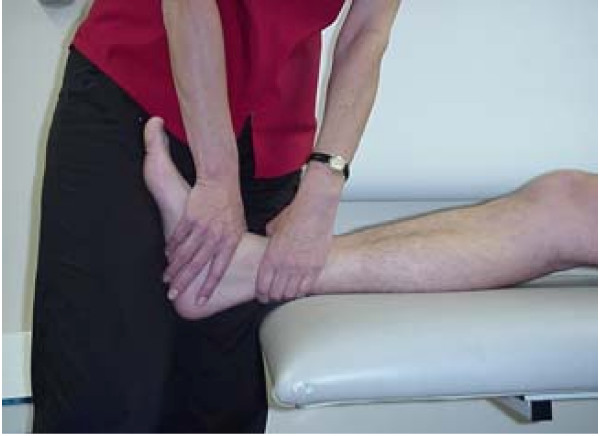
Passive joint mobilisation. The joint mobilisation technique used will be the anterior-posterior glide of the talus. The physiotherapist will apply large amplitude oscillatory movements into resistance (i.e., Grade III) on the talus.

Participants in the *treatment group *will receive passive joint mobilisation (see Figure 2). The joint mobilisation technique used will be the anterior-posterior glide of the talus [23]. The participant will be positioned in supine or long sitting, with the affected ankle moved to the end of the available range of pain-free dorsiflexion. The physiotherapist will manually apply large amplitude oscillatory movements into resistance (i.e., Grade III [23]) in an anterior-posterior direction on the talus for three sets of 60 seconds. This can be progressed by increasing the force and repetitions (up to five sets of 60 seconds) of the mobilisation, and the degree of ankle dorsiflexion. The choice of this technique is based on findings of a previous study, which showed that the anterior-posterior glide of the talus was more effective than usual care after ankle sprain [35].

**Figure 3:**
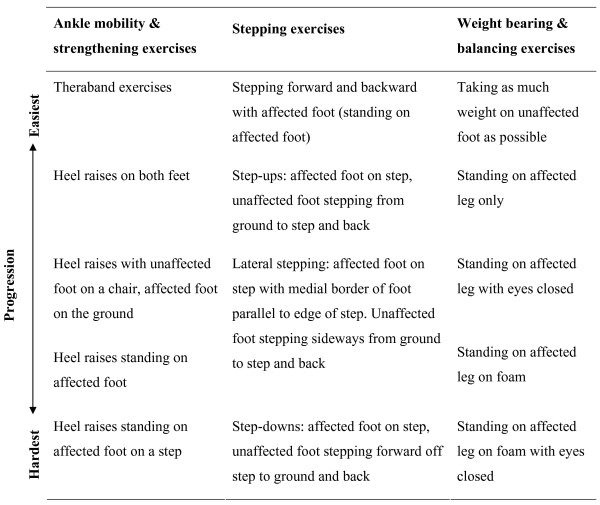
The home exercise programme. Participants will perform one exercise from each category of exercise. The physiotherapist will be responsible for the prescription, instruction, monitoring, and progression of the exercises.

Participants in both the *treatment and control groups *will be given an exercise programme that they will continue at home. Exercises will be chosen from a standardised set of exercises to ensure consistency. The exercises have been developed following consultation with physiotherapists at the recruiting hospitals and are based on the programme used in a previous study [41]. Three categories of exercise will be prescribed: ankle mobility and strengthening exercise, stepping exercise, and weight bearing and balancing exercise (see Figure 3). Participants will perform one exercise from each category at any one time. The treating physiotherapist will be responsible for the progression of the exercise programme, so the exercises will be tailored to the individual participant's needs and will always be challenging and difficult when repeated 10 times. Participants will be given exercise cards and exercise recording sheets to record the number of repetitions performed each day. The recommended frequency for most exercises will be three sets of 10 repetitions per exercise per day.

In this pragmatic trial, participants will be seen at a frequency typical to that which occurs in the clinical setting. Participants in the treatment group will receive two treatment sessions per week for four weeks for joint mobilisation and progression of the exercise programme. Participants allocated to the control group will receive two treatment sessions in the first week, then one session per week for three weeks. In the first session, participants in the control group will be taught the exercises, which will be monitored and progressed in subsequent sessions.

In addition, at the treatment sessions all participants will receive gait retraining, progression of walking aids, and advice on prognosis and return to activities. If required, participants will receive ice for pain relief, and compression and elevation for the management of swelling. No other physiotherapy treatments will be administered during the four-week intervention period.

After each initial treatment session, the treating physiotherapist will receive a phone call from an investigator unblinded to group allocation. Implementation of the experimental protocol for each participant will be discussed to ensure compliance.

At the end of the intervention period, passive joint mobilisation treatment will cease and participants will be allowed to be progressed to exercises other than those in the standardised set. Ankle taping can be performed to assist return to sports.

The timing of discharge will not be dictated in the protocol. Participants will be discharged by their physiotherapists when they return to their previous level of function, reach a plateau in their progress, or choose to discontinue treatment.

### Outcome assessment

Participants will be assessed at baseline and the four, 12, and 24 week follow-up sessions by an assessor blinded to group allocation. The physiotherapists, participants and investigators will be asked to avoid giving information which may reveal group allocation to the blinded assessor. Assessor blinding will be checked after each follow-up assessment. The assessor will be asked if he/she was unblinded and will guess the allocation of each participant.

Demographic information will be collected and an X-ray assessment will be carried out by the blinded assessor at the baseline assessment. The X-ray assessment will determine the angle at which the fractured ankle was immobilised and the number of malleoli involved, in order to investigate the relationships between the angle of immobilisation and fracture severity with outcome.

The primary outcomes will be activity limitation and quality of life. They will be measured by the Lower Extremity Functional Scale [42] and the Assessment of Quality of Life scale [43], respectively. On the Lower Extremity Functional Scale, participants will rate the difficulty they have in performing 20 activities on a five-point scale from zero (extreme difficulty or unable to perform activity) to four (no difficulty). The maximum score is 80 points and a higher score denotes less limitation in activity. When tested in a sample of participants with lower limb injuries that included ankle fracture, the scale showed high internal consistency (alpha = 0.96) and test-retest reliability (intraclass correlation coefficient = 0.86), and correlated well with the physical component of the Short Form 36 [42]. The Lower Extremity Functional Scale is capable of discriminating between patients requiring walking aides and patients not requiring walking aides, and has moderate correlations with weight-bearing status and ankle movement [44].

The Assessment of Quality of Life is designed to measure health-related quality of life, and has 15 questions covering five dimensions: illness, independent living, social relationships, physical senses and psychological wellbeing [43]. It has high internal consistency (alpha = 0.81) [43]. The Assessment of Quality of Life is sensitive to changes in health states and is highly correlated with other commonly-used quality of life scales [46]. Population norms have been calculated for the Australian population [46].

Twelve secondary outcome measures will be used (Table 1). All participants will be given a calendar on which to mark the first day they can walk pain-free for 10 metres at a comfortable cadence. This will be used to calculate the number of days to pain-free walking from cast removal.

Walking speed and step length asymmetry will be measured as the participants walk unaided along a 14-metre walkway. The average of three attempts will be used for data analysis. Participants will be instructed to walk as fast and as well as possible. Their time to walk the central 10 metres will be recorded by a stop watch, and walking speed will be calculated by dividing the distance covered by the time elapsed. A six-metre long, one-metre wide mat marked with five-centimetre grids will be taped to the floor over the central six metres of the walkway. Participants will be videotaped as they walk across the mat. Step length asymmetry (i.e., the difference in step length between the affected and unaffected sides) will be calculated by playing the video recording in slow motion, and digitising the heel position of each footfall on a customised computer programme [47]. This method has high inter-rater, intra-rater and concurrent reliability [47-50].

Stepping rate on stairs will be calculated by the participants climbing four steps three times as fast as possible without using handrails. This task is part of the Motor Assessment Scale [51]. Although its psychometric properties have not been examined in people with musculoskeletal complaints, stair climbing will be included as an outcome measure as it is reasonably challenging and is a commonly-performed activity.

Ankle dorsiflexion range of motion will be measured using the weight-bearing lunge method [52]. Participants will stand with the affected foot on the ground and the great toe against the wall, and will bend the knee to touch the wall. If the knee cannot reach the wall, the minimum distance between the knee and the wall without the heel lifting will be recorded in millimetres as a negative value. If the knee can touch the wall, the foot will be gradually moved back and the maximum distance between the great toe and the wall without the heel lifting will be recorded in millimetres as a positive value. Measured this way, ankle dorsiflexion at the time of cast removal has been shown to be a significant predictor of disability, perceived change in symptoms and active ankle dorsiflexion range six weeks and six months after ankle fracture [14]. The weight bearing lunge method has high inter-rater and intra-rater reliability (intraclass correlation coefficient 0.99 and 0.97 to 0.98 respectively) in the healthy population [52].

Pain on standing with equal weight distribution and during stair descent will be measured using a 100-mm visual analogue scale, labelled "no pain" on one end and "worst pain ever" on the other. The pain visual analogue scale correlates positively with other self-reported measures of pain intensity [53,54]. It has high test-retest reliability (intraclass correlation coefficient = 0.71 to 0.99) [53], and is highly responsive in detecting change [54]. Participants will also rate their return to usual work, and return to usual sport and leisure activities using a 100-mm visual analogue scale, marked "not participating at all" at one end and "return to full level" at the other.

Global perceived effect of treatment will be measured on an 11-point scale, where participants rate their change in symptoms from -5 (vastly worse) to +5 (completely recovered). A scale of this kind is the most frequently used external criterion to which score changes on other outcome measurements are compared [42,55-57]. However its use as an external criterion is under debate [58,59], and there is little information regarding its validity and reliability [59].

Satisfaction with physiotherapy treatment will be measured at the four-week follow-up assessment on a 100-mm visual analogue scale marked "completely unsatisfactory" at one end and "best possible" at the other. At all follow-up assessments, participants will be asked if the physiotherapy treatment has had any negative effects and, if so, the nature of the effects. They will also record whether they have had treatment other than physiotherapy for the fractured ankle.

The treating physiotherapist will complete a survey for every participant at each follow-up assessment until discharge. Information gathered on this survey will be: the date and reason of discharge (if applicable), number of physiotherapy sessions scheduled, number of physiotherapy sessions attended, exercises prescribed and repetitions completed, any other physiotherapy treatment administered, and, for the treatment group only, the dates, number of sets and duration of the joint mobilisation treatment. This information will be analysed to obtain the length of physiotherapy required, percentage of scheduled sessions attended, compliance of participants to the exercise programme, and compliance of physiotherapists to the experimental protocol.

Data will be collected on paper forms and manually entered into a computer programme. Several strategies will be implemented to ensure the accuracy of the data. Data collection forms will be checked for completion. Data entry will be performed twice and the two entries will be compared to check for errors. Range checks will be performed on outcome values at the completion of data entry.

### Economic analysis

**Figure 4:**
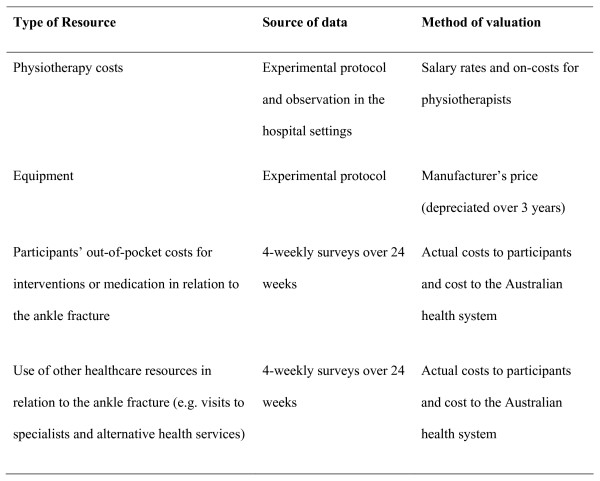
Economic evaluation

The economic analysis will be conducted from the perspective of the Australian healthcare system and the individual patient, with data collected in a survey every four weeks from the end of the four-week intervention period to the 24-week follow-up assessment. Participants will record all out-of-pocket expenses incurred due to the fractured ankle, including the frequency and costs associated with physiotherapy, other health and hospital services, and the costs of medication. Figure 4 indicates the type of resources which will be captured, the sources of data, and proposed methods of valuation. At the four-week follow-up assessment, participants will also be asked to provide socio-demographic information such as their usual main activity, normal annual household income, the type of private health insurance they have (if any), and whether they are renting, boarding or owners of their homes.

### Statistical analysis

Data analysis will be undertaken at the completion of data acquisition and will be by intention to treat (that is, participants will be analysed according to group allocation regardless of compliance) [60]. Missing data will be replaced by the last known value carried forward. The statistician will be given coded data to ensure blinding.

Separate analyses will be performed on the four, 12 and 24 week data, with the four-week (i.e., the end of the intervention period) follow-up being the primary time point for analysis. To test the effects of treatment, between-group differences will be examined with analysis of covariance using a regression approach. The emphasis will be on estimation, but hypothesis tests will also be conducted (alpha = 0.05). Pre-test scores will be entered into the model as the only covariate.

In a secondary analysis designed to test the influence of fracture severity on treatment effects, additional terms (fracture severity, group by fracture severity interaction) will be entered into the regression model. Fracture severity will be categorised into two levels based on the number of malleoli involved: less severe (unimalleolar fractures) and more severe (bimalleolar or trimalleolar fractures). The effect of fracture severity on treatment will be determined by examining the interactions between group of allocation and fracture severity.

Ninety participants (45 per group) will participate in the trial. This sample size provides a 90% probability of detecting a difference between group means of 10 points on the Lower Extremity Functional Scale, assuming a standard deviation of 12 based on a recently published study [41]. These calculations assume a correlation of 0.6 between pre- and post-test measures and an alpha of 0.05, and allowed for 10% dropouts and 20% non-compliance.

The economic evaluation will consist of cost-effectiveness and cost-utility analyses. The cost-effectiveness analysis will use the Lower Extremity Functional Scale as a measure of effectiveness. The cost-utility analysis will use the Assessment of Quality of Life as a measure of utility [43]. The analyses will examine differences between treatment and control participants in terms of costs incurred and reductions in perceived disability (cost-effectiveness analysis) or utility gained (cost-utility analysis).

The incremental cost-effectiveness (utility) ratio (ICER) will be calculated as: ICER = (C_E _- C_C_)/(U_E _- U_C_), where C is average cost, U is the average effectiveness or utility score, and subscripts E and C denote the treatment and control arms. Joint mobilisation can be said to be cost-effective relative to exercise alone if it produces less perceived disability and/or greater utility at a lower cost, or the cost per disability avoided or per quality of life gained (i.e., the ICER) is less than some threshold value (e.g., $50,000 in Australian dollars). Sensitivity analysis will be undertaken to explore the robustness and validity of the results. That is, both costs and outcomes will be varied in line with results from similar studies reported in the literature and the upper and lower bounds of the results from this trial.

### Funding

The trial is funded by the Motor Accidents Authority of New South Wales, Australia (grant reference number 04/241).

## Discussion

The primary aim of this randomised controlled trial is to determine the effectiveness and cost-effectiveness of passive joint mobilisation, in addition to an exercise programme, in adults after cast immobilisation for ankle fracture. The trial incorporates design features known to minimise bias [62]. Participants will be assigned to treatment or control groups using a concealed random procedure. Assessments will be blinded. Data analysis will also be blinded and by intention to treat.

Blinding confers several potential benefits to the rigor of randomised controlled trials, particularly minimising bias [61,62]. Failure to implement blinding could lead to inflated estimates of effects [62,63]. The nature of the treatments involved in this trial means that blinding of the physiotherapists and participants is not possible. Nevertheless, satisfaction with physiotherapy will be evaluated as a means to assess participants' perception of the treatment received.

A pragmatic trial design has been chosen as it can directly inform clinical practice [64, 65, 66, 67]. The inclusion criteria of the trial reflect the variety in patient presentations that would be encountered by physiotherapists in the clinical setting. The experimental protocol also simulates clinical practice. Participants will be treated with the same frequency and for the same duration as would be normal practice, and one form of treatment (joint mobilisation plus exercise) will be compared to another form of treatment (exercise only), instead of placebo. The outcome measures chosen are clinically oriented and participants will be analysed according to group allocation regardless of compliance. Using a pragmatic design broadens the generalisability of the results. An added advantage to this trial is that the pragmatic design allows a realistic economic evaluation of the two treatment alternatives [65].

It is anticipated that data acquisition will finish by early 2007. Results from this trial will contribute to evidence-based guidelines for rehabilitation after ankle fracture.

## Competing interests

The author(s) declare that they have no competing interests.

## Authors' contributions

AM, KR, MH and RH conceived and designed the trial protocol, and procured funding. MH designed the economic analysis. RH designed the statistical analysis. CL refined and developed the trial protocol. CL implemented the trial protocol, managed the trial, performed blinded outcome assessment and data entry, and drafted the manuscript. AM, KR, MH and RH contributed to the manuscript. All authors read and approved the final manuscript.

## Pre-publication history

The pre-publication history for this paper can be accessed here:



## Figures and Tables

**Table 1:** Secondary outcome measures

Number of days to pain-free walking	
Measures of activity limitation:	Unaided walking speed
	Step length asymmetry during unaided walking
	Stepping rate on stair climbing
Measures of impairments:	Ankle dorsiflexion range of motion
	Pain on standing with equal weight distribution
	Pain on stair descent
Measures of participation:	Return to usual work
	Return to sport and leisure activities
Global perceived effect of treatment	
Satisfaction with physiotherapy treatment	
Negative effects of physiotherapy treatment	
